# The socioeconomic and lifestyle determinants of contraceptive use among Chinese college students: a cross-sectional study

**DOI:** 10.1186/s12978-020-00978-9

**Published:** 2020-08-17

**Authors:** Yuqi Wang, Minne Chen, Shihui Tan, Xueqi Qu, Hanyu Wang, Xiao Liang, Junjian Gaoshan, Lihe Li, Ping Hong, Li Jiang, Kun Tang

**Affiliations:** 1 Vanke School of Public Health, Tsinghua University, Zhongguancun North Street, Haidian District, Beijing, 100084 China; 2 School of Health Humanities, Peking University, No 38 Xueyuan Rd, Haidian District, Beijing, 100191 China; 3 University of North Carolina at Chapel Hill, Chapel Hill, NC 27599 USA; 4 School of Basic Medical Sciences, Peking University, No 38 Xueyuan Rd, Haidian District, Beijing, 100191 China; 5 School of Public Health, Johns Hopkins University, Baltimore, MD 21218 USA; 6 China Family Planning Association, Building 35, Shaoyaoju Chaoyang District, Beijing, 100029 China; 7 United Nations Population Fund China, Liangmahe Road, Chaoyang District, Beijing, 100029 China; 8 Peking University People’s Hospital, 11 Xizhimen South Street, Xicheng District, Beijing, 100044 China

**Keywords:** Sexual behaviours, Contraception, Socioeconomic and lifestyle determinants, Chinese youth

## Abstract

**Background:**

Chinese youth have become more sexually active over the years, yet their behaviours of contraceptive use are influenced by socioeconomic and lifestyle factors. This study aimed to investigate the socioeconomic and lifestyle determinants of contraceptive use among Chinese college students, and to investigate quantitatively their reasons for choosing different contraceptive methods.

**Methods:**

The study used the data from a cross-sectional survey, which was conducted in 2015 among 17,517 students from 130 colleges and professional schools in mainland China. Chi-square tests were performed to test the differences in the awareness and uses of contraceptives between male and female students. Multivariable logistic regression models were used to analyse the associations between socioeconomic and lifestyle factors and contraceptive uses.

**Results:**

Condoms (95%), oral contraceptives (91%) and emergency contraceptives (71%) were the most commonly known contraceptive methods among the sample of Chinese youth that were surveyed. Among male participants, high monthly expenditure (OR = 1.37, 95%CI: 1.07–1.75), light alcohol consumption (OR = 1.47, 95%CI: 1.03–2.11), and high sexual knowledge (OR = 1.96, 95%CI: 1.57–2.45) were positively associated with contraceptive uses, while tobacco use (OR = 0.47, 95%CI: 0.23–0.95) showed a negative association with contraceptive uses. Among female students, suburban residency (OR = 1.38, 95%CI: 1.03–1.83), high level of parental education (OR = 1.96, 95%CI: 1.19–3.22), moderate alcohol consumption (OR = 2.66, 95%CI: 1.05–6.77), and high sexual knowledge (OR = 1.50, 95%CI: 1.18–1.91) were positively associated with contraceptive uses, while tobacco use (OR = 0.60, 95%CI: 0.36–0.99) showed a negative association.

****Conclusion**s:**

A series of socioeconomic and lifestyle factors were associated with contraceptive choices among Chinese college students. Targeted sexual education programs are in demand to improve the awareness and the use of contraceptives in this population.

## Plain English summary

As Chinese youth are becoming more sexually active over the years, it is increasingly important to understand the potential determinants of contraceptive uses in this population. In this study, we investigated the contraceptive use and the awareness of contraception of Chinese students based on a survey conducted in 2015. Condoms, oral contraceptives and emergency contraceptives were the most known methods among Chinese college students. Most students reported using condoms or withdrawal method during their last sexual intercourse. Tobacco use and lack of reproductive health knowledge might discourage participants’ uses of contraceptives, while suburban residency, high monthly expenditure, high parental level of education, light alcohol consumption and high reproductive health knowledge score encouraged the use of contraceptives among both male and female participants. Chinese college students generally prioritized the practicality, the efficacy, and having minimal side effects when choosing among different contraceptive methods. In order to improve the awareness and the use of contraceptives in this population, targeted sexual education programs are in demand in mainland China.

## Background

Risky sexual behaviours increase the prevalence of negative sexual and reproductive (SRH) outcomes, such as unintended pregnancy, induced abortion, and sexually transmitted infections (STIs) [1]. It is well established that appropriate contraception is an effective way to prevent these outcomes [2]. Meanwhile, most of the unwanted pregnancies were due to improper use of contraception and lack of contraceptive information, posing serious physical and psychological health issues in young people, and causing significant social burdens [3, 4].

With economic reforms and profound social changes, attitudes towards pre-marital sex among Chinese young people are no longer as conservative as before. Chinese youth had gradually become more sexually active [4, 5]. A study in 2013 showed that about 24% of undergraduates had experienced premarital sexual intercourse [6]. This was an approximately 4-fold increase than reported in 2002 [7]. In terms of contraceptive use, however, Chinese youth seemed to overlook its importance. A study in seven Chinese cities showed that the most popular contraceptive methods among college students were condoms and oral contraceptives [8]. However, there were still about a third of the college students that did not adopt any contraceptive methods in their last sexual intercourse [9]. In terms of the knowledge of contraception, up to 30% Chinese students were not aware of the importance of contraception, which foreboded an increasing number of high-risk sexual behaviours [5, 6]. Among students reporting premarital sexual intercourse, about 20% of them acknowledged unintended pregnancy, which resulted in a high prevalence of induced abortion [6].

Despite the increasing accessibility of various contraceptive methods, especially condoms, in China, the contraceptive use among college students was still low [8]. There were several studies exploring the factors associated with abortion and other adverse sexual outcomes [5, 8–10], yet the associations between socioeconomic and lifestyle factors and the contraceptive preference among college students were seldom discussed. This study aimed to analyse the awareness, the preference, and the prevalence of contraceptive use among Chinese college students, and to identify the socioeconomic and lifestyle determinants of contraceptive use among college students in China.

## Methods

### Study sample

The data was collected from an Internet-based survey that was distributed to 20,088 undergraduates from 130 colleges and professional schools in mainland China in 2015. The survey aimed to investigate SRH knowledge and behaviours among Chinese college students. Convenient sampling was used to select universities and colleges. Among the 130 participating schools, 61.6% were located in eastern China, 24.8% in central China, and 13.6% were in western China. Given the unbalanced distribution of educational resources, the proportions were corresponding to the overall distribution of colleges and universities in mainland China [11]. The link to the questionnaire was given to contact persons from each selected college. The contact persons helped to assign the survey to one or two randomly selected classes based on the total number of students in that particular college. Identifiable information was not included to ensure the privacy of the respondents, and informed consent was obtained from the respondents prior to them entering the survey. IP address of each submission was recorded to avoid repetitive submissions. Participants were included if they: 1) were 18–25 years’ old; 2) were Chinese undergraduates enrolled in colleges or universities in mainland China; 3) answered all required questions. Self-identified homosexual participants were excluded from the current analysis, as they were not directly affected by issues of unwanted pregnancy. Moreover, many contraceptive methods such as pills, injectable contraceptives and oral emergency contraceptives,^1^ were not relevant in this population. In total, the overall response rate was 89%, and 17,517 students were included in the final analyses. Schools from which the participants were from were included in the analyses to adjust for clustering.

### Measures and variables

The reproductive health questionnaire consisted of three main parts: 1) baseline information including age, sex, type of school, year of enrolment, major, monthly expenditure, hometown, parental level of education, and smoking and drinking behaviors; 2) sexual and reproductive health knowledge; 3) sexual attitudes and sexual behaviors. In this study, baseline characteristics, past exposure to sexual education, SRH knowledge, and sexual behaviours were included in the analyses. The type of school enrolled was categorized into two groups: colleges and professional schools. Monthly expenditure was collected from the self-reported survey, and was coded into three categories: less than 1000RMB exclusive, between 1000 and 2000RMB inclusive, and more than 2000RMB exclusive (1 USD ≈ 6.3 RMB). Hometown referred to the place one lived in before he or she entered college, and was coded into three categories: rural, suburban, and urban. Parental level of education was coded as the higher educational attainment of either parents. It included three categories: primary school and below, middle/high school, and vocational school or above. Lifestyle factors included tobacco use and alcohol consumption. Alcohol consumption was measured based on the information on types of alcohol, the amount and the frequency of drinking. Drinking habits were coded into four categories: abstinence, light, moderate, and heavy, and the method of classification is shown in Additional file 1. Participants’ previous exposure to sexual knowledge was assessed using a short SRH quiz. The quiz included topics on contraception, HIV/AIDS, pregnancy/abortion, and masturbation. The score of the quiz ranged between 0 and 4 according to the number of the correct answers attained.

The main outcome variables of this study were participant’s awareness of contraceptive methods and the use of contraceptive method(s) in participant’s last sexual intercourse. For the awareness of contraceptives, participants were asked if they had heard of the listed contraceptive methods. The list of contraceptive methods included ‘none’, ‘spermicide’, ‘withdrawal’, ‘rhythm method’, ‘condom’, ‘oral contraceptives’, ‘injectable contraceptives’, ‘emergency contraceptive (EC)’, ‘intrauterine devices (IUD)’, ‘male/female sterilization’ and ‘others’. Participants were asked to choose from the same list of contraceptive methods, except ‘IUD’ and ‘male/female sterilization’ when assessing the contraceptive method(s) used during their last sexual intercourse, since male/female sterilization and IUDs were rarely used among Chinese college students.

Reasons for using the particular contraceptive method(s) chosen by the participants were also recorded. Participants were asked to choose from a list of reasons, including ‘sexual partner’s request’, ‘accessibility’, ‘practicality’, ‘effectiveness’, ‘few side effects’, ‘little influence on sexual sensation during intercourse’, and ‘others’. Participants who did not use any contraceptives in the last sexual intercourse were asked to choose from the following possible reasons: ‘sexual partner’s request’, ‘no need’, ‘poor sexual experience’, ‘inaccessibility’, ‘difficulties in using’, ‘side effects’, ‘inefficacy’ and ‘others’. The list of possible reasons were drawn in reference to a previous study [8].

### Data analysis

Descriptive statistics illustrates the basic characteristics of our participants. Cross tabulations and Pearson’s χ2 tests were used to compare the differences in contraceptive awareness and the prevalence of contraceptive use between males and females. The current study also analysed participants’ reasons for using and not using a particular contraceptive. Multivariable logistic regression models were used to assess the associations of socioeconomic (i.e. type of school, sex, monthly expenditure, hometown, parental level of education), lifestyle factors (i.e. tobacco use, alcohol consumption) and sexual education with self-reported contraceptive use in the last sexual intercourse. Logistic regression models were also used to analyse the socioeconomic and lifestyle factors associated with specific contraceptive choices (i.e. oral/injectable contraceptives, condoms, and EC). All analyses were done using Stata 14.0 (Stata Corp, Tex, USA) with a significance level of 0.05.

## Results

### Characteristics of the participants

The characteristics of the study participants were presented in Table 1. In total, 17,517 participants were included in this study, and 72% of them were from colleges. Among all participants in the sample, 3428 (20%) of them reported previous sexual experience. The mean age (SD) of the participants was 20.18 (1.22) years, and males accounted for 39% of participants. About 56% of the participants had previously received some form of sexual education.

**Table 1:** Characteristics of the survey population (%)

	Never had sex	Had sex before	Total
	***n*** = 14,089	***n*** = 3428	***n*** = 17,517
**Socio-demographic characteristics**			
Age			
< 20	33	22	31
20–21	56	56	56
≥ 22	11	22	13
Male	35	54	39
**Education**			
School			
College	72	73	72
Professional School	28	27	28
Year			
Freshman	48	33	45
2nd year	35	36	35
3rd year	14	23	16
4th year and older	4	8	5
**Expenditure per month (RMB)**			
< 1000	53	36	50
1000–2000	41	50	43
> 2000	6	14	7
**Hometown**			
Urban	38	46	39
Suburban	32	29	31
Rural	31	24	29
**Parental education**			
Primary school and below	10	10	10
Middle and high school	66	61	65
Vocational school and above	24	29	25
**Lifestyle**			
Tobacco use			
No smoking	95	77	91
1–10 cigarettes/day	4	17	7
> 10 cigarettes/day	1	6	2
Alcohol consumption			
Abstinence	33	17	30
Light	58	59	59
Moderate	6	15	8
Heavy	2	9	4
**Exposure to sexual education**			
Ever received any kind of sexual education	56	53	56

### Awareness and use of contraceptive methods

The awareness and the prevalence of contraceptive use were presented in Table 2. Both male and female students were highly aware of condom (95% vs. 96%, *p* = 0.19), oral contraceptives (90% vs. 91%, *p* < 0.01), and emergency contraception (70% vs. 72%, *p* < 0.01), followed by withdrawal (58% vs. 43%, *p* < 0.01), rhythm method (56% vs. 53%, *p* < 0.01), female and male sterilization (46% vs. 48%, *p* = 0.01; 45% vs. 45%, *p* = 0.56), and intrauterine devices (31% vs. 33%, *p* = 0.01). During the last sexual intercourse, participants tended to use condoms (63%), use withdrawal (17%), or use no method (15%), while few students chose oral contraceptives (4%), ECs (4%), or rhythm method (5%). Despite of the high awareness of oral contraceptives, ECs, or rhythm method, the prevalence of these methods was not as high.

**Table 2:** Awareness and use of different contraceptive methods among Chinese college students

	Awareness (%)				Contraceptive use during last sex	
	Male	Female	χ2	Pr^b^	n	%
	*n* = 6827	*n* = 10,690				
Never heard of any method	1	1	1.34	0.25	-^a^	-^a^
No contraceptive use	-^a^	-^a^		-^a^	530	15
Spermicide	13	9	91.88	< 0.01	-^a^	-^a^
Withdrawal	58	43	355.54	< 0.01	587	17
Rhythm method	56	53	19.69	< 0.01	158	5
Condom	95	96	1.75	0.19	2169	63
Oral contraceptive	86	91	12.85	< 0.01	137	4
Injectable contraceptive	19	14	82.95	< 0.01	31	1
Emergency contraception	70	72	9.79	< 0.01	141	4
Intrauterine device (IUD)	31	33	6.22	0.01	-^a^	-^a^
Sterilization						
Male	45	45	0.30	0.59	-^a^	-^a^
Female	46	48	6.28	0.01	-^a^	-^a^
Others	1	1	0.16	0.69	100	3

^a^No data

^b^Pr for chi-square test

### Associations between socioeconomic and lifestyle factors and contraceptive use

The associations of demographic, socioeconomic, and lifestyle factors with contraceptive use among male and female participants are presented in Table 3 and Table 4. In terms of socioeconomic factors, having a monthly expenditure of 1000 ~ 2000 RMB was positively associated with self-reported contraceptive use among male students (OR = 1.37, 95%CI: 1.07–1.75). In particular, male participants with a monthly expenditure of 1000-2000RMB were more likely to use condoms (OR = 1.48, 95%CI: 1.16–1.89). Among female participants, students whose parents had received middle/high school education and vocational school or higher education were more likely to use condoms (OR = 1.75, 95%CI: 1.16–2.65; OR = 1.94, 95%CI: 1.20–3.15). Female students from suburban areas were more likely to use contraceptive methods, compared to participants from urban areas (OR = 1.38, 96%CI: 1.03–1.83).

**Table 3:** Logistic regression models examining the odds of contraceptive methods used during last sexual intercourse among male participants

	Specific contraceptive methods used							
	Any self-reported use of contraception		Oral & injectable contraceptives		Condom		Emergency contraceptives	
	*n*^a^ = 1177		*n*^a^ = 101		*n*^a^ = 1174		*n*^a^ = 61	
	OR	95% CI	OR	95% CI	OR	95% CI	OR	95% CI
School								
College	1.00		1.00		1.00		1.00	
Professional School	0.79	0.54–1.18	0.87	0.33–2.31	0.98	0.66–1.45	0.50	0.16–1.58
*P for difference*	0.26		0.79		0.92		0.24	
Geographical distribution								
Eastern	1.00		1.00		1.00		1.00	
Central	0.65	0.32–1.32	2.83	0.61–12.99	0.62	0.32–1.21	4.15	0.65–26.66
Western	0.71	0.38–1.32	1.82	0.37–8.58	1.06	0.57–2.00	1.92	0.40–9.06
*P for difference*	< 0.01		0.13		< 0.01		0.55	
Expenditure per month								
Less than 1000	1.00		1.00		1.00		1.00	
1000–2000	1.37*	1.07–1.75	0.83	0.50–1.39	1.48**	1.16–1.89	0.98	0.50–1.94
More than 2000	1.03	0.72–1.47	0.63	0.27–1.48	1.12	0.78–1.61	1.06	0.41–2.72
*P for trend*	0.37		0.29		0.17		0.90	
Hometown								
Urban	1.00		1.00		1.00		1.00	
Suburban	1.23	0.95–1.60	1.59	0.93–2.73	1.05	0.81–1.35	0.95	0.46–1.96
Rural	1.15	0.86–1.54	0.93	0.48–1.80	1.02	0.76–1.37	0.97	0.45–2.12
*P for difference*	0.28		0.13		0.28		0.73	
Parental education								
Primary school and below	1.00		1.00		1.00		1.00	
Middle & high school	0.89	0.63–1.27	0.85	0.42–1.75	1.03	0.73–1.45	0.99	0.39–2.49
Vocational school and higher	0.91	0.61–1.36	1.37	0.60–3.11	0.92	0.62–1.37	1.02	0.34–3.09
*P for difference*	0.926		0.64		0.61		0.80	
Tobacco use								
No smoking	1.00		1.00		1.00		1.00	
1–10 cigarettes/day	0.93	0.72–1.19	0.94	0.55–1.60	1.06	0.82–1.37	0.47*	0.23–0.95
> 10 cigarettes/day	0.91	0.62–1.34	1.28	0.60–2.74	0.68	0.46–1.00	0.83	0.32–2.13
*P for trend*	0.45		0.71		0.17		0.25	
Alcohol consumption								
Abstinence	1.00		1.00		1.00		1.00	
Light	1.41	0.98–2.05	0.65	0.32–1.31	1.47*	1.03–2.11	0.72	0.30–1.69
Moderate	1.31	0.86–1.99	0.76	0.36–1.62	1.41	0.94–2.12	0.85	0.21–2.32
Heavy	1.18	0.75–1.86	0.82	0.32–2.07	1.41	0.91–2.21	0.49	0.14–1.78
*P for trend*	0.90		0.93		0.40		041	
Sexual knowledge								
SRH knowledge score < =2	1.00		1.00		1.00		1.00	
SRH knowledge score > 2	1.96*	1.57–2.45	0.72	0.44–1.15	1.90**	1.52–2.37	1.13	0.62–2.05
*P for difference*	< 0.01		0.17		< 0.01		0.69	

^a^The n refers to the number of participants who reported specific contraceptive use during the last sexual intercourse. The total number of participants analysed via logistic model is 1855

^b^Adjustments: type of school, school, geographical distribution, expenditure per month, hometown, parent education, tobacco use, alcohol consumption, sex education

* *P* < 0.05, ** *P* < 0.01

**Table 4:** Logistic regression models examining the odds of contraceptive methods used during last sexual intercourse among female participants

	Specific contraceptive methods used							
	Any self-reported use of contraception		Oral & injectable contraceptives		Condom		Emergency contraceptives	
	*n*^a^ = 1019		*n*^a^ = 55		*n*^a^ = 995		*n*^a^ = 80	
	OR	95% CI	OR	95% CI	OR	95% CI	OR	95% CI
School								
College	1.00		1.00		1.00		1.00	
Professional School	0.72	0.44–1.18	0.67	0.22–2.09	0.79	0.49–1.28	1.38	0.46–4.17
*P for difference*	0.20		0.50		0.49		0.57	
Geographical distribution								
Eastern	1.00		1.00		1.00		1.00	
Central	1.16	0.50–2.66	0.68	0.12–3.82	1.03	0.47–2.27	0.57	0.12–2.69
Western	0.83	0.37–1.88	0.80	0.16–3.92	1.11	0.48–2.52	0.48	0.09–2.50
*P for difference*	0.68		0.88		0.53		0.10	
Expenditure per month								
Less than 1000	1.00		1.00		1.00		1.00	
1000–2000	1.01	0.77–1.32	0.71	0.36–1.39	1.14	0.87–1.48	1.08	0.60–1.93
More than 2000	1.08	0.71–1.63	1.20	0.50–2.83	1.11	0.74–1.65	2.19*	1.07–4.48
*P for trend*	0.86		0.93		0.70		0.06	
Hometown								
Urban	1.00		1.00		1.00		1.00	
Suburban	1.38*	1.03–1.83	0.94	0.44–1.98	1.11	0.84–1.48	1.15	0.63–2.12
Rural	1.15	0.81–1.62	1.13	0.46–2.77	1.02	0.73–1.43	1.10	0.54–2.26
*P for difference*	0.04		0.64		0.02		0.83	
Parental education								
Primary school and below	1.00		1.00		1.00		1.00	
Middle & high school	1.81	1.18–2.78	1.16	0.36–3.77	1.75**	1.16–2.65	1.32	0.51–3.41
Vocational school and higher	1.96	1.19–3.22	1.64	0.47–5.74	1.94**	1.20–3.15	0.91	0.31–2.63
*P for difference*	< 0.01		0.57		< 0.01		0.58	
Tobacco use								
No smoking	1.00		1.00		1.00		1.00	
1–10 cigarettes/day	0.61	0.36–1.00	0.85	0.21–3.51	0.60*	0.36–0.99	1.72	0.77–3.84
> 10 cigarettes/day	0.32	0.11–0.95	2.86	0.50–16.31	0.36	0.12–1.09	2.35	0.27–20.76
*P for trend*	< 0.01		0.29		< 0.01		0.16	
Alcohol consumption								
Abstinence	1.00		1.00		1.00		1.00	
Light	1.16	0.90–1.51	0.73	0.39–1.37	1.22	0.94–1.58	1.13	0.60–2.13
Moderate	1.45	0.83–2.52	1.21	0.37–3.97	1.07	0.64–1.78	2.66*	1.05–6.77
Heavy	1.38	0.56–3.43	0.55	0.02–13.29	1.10	0.46–2.67	2.08	0.52–8.26
*P for trend*	0.14		0.60		0.37		0.09	
Sexual knowledge								
SRH knowledge score < =2	1.00		1.00		1.00		1.00	
SRH knowledge score > 2	1.50*	1.18–1.91	0.56	0.30–1.01	1.23	0.96–1.56	1.55	0.89–2.69
*P for trend*	< 0.01		0.06		0.10		0.12	

^a^The n refers to the number of participants who reported specific contraceptive use during the last sexual intercourse. The total number of participants analysed via logistic model is 1573

^b^Adjustments: type of school, school, geographical distribution, expenditure per month, hometown, parent education, tobacco use, alcohol consumption, sex education

* *P* < 0.05, ** *P* < 0.01

In terms of lifestyle factors, tobacco use was negatively associated with contraceptive uses, while alcohol consumption presented a positive association. By further analysing the association between different contraceptive methods and lifestyle factors, we found that among male population, having over 10 cigarettes per day was negatively associated with condom use (OR = 0.68, 95%CI: 0.46–1.00). Meanwhile, having 1–10 cigarettes per day was negatively associated with ECs (OR = 0.47, 95%CI: 0.23–0.95). Among female participants, having 1–10 cigarettes per day was negatively associated to overall contraceptive use (OR = 0.32, 95%CI: 0.11–0.95), as well as condom use in specific (OR = 0.60, 95%CI: 0.36–0.99). Alcohol consumption was positively associated with condom use in male population, especially within the group of light drinkers (OR = 1.47, 95%CI: 1.03–2.11). For females, moderate alcohol consumption was positively associated with ECs (OR = 2.66, 95%CI: 1.05–6.77). Additional regression results for withdrawal method showed that neither smoking nor drinking habit was significantly associated with withdrawal method for both males and females (Additional file 3).

Participants’ SRH knowledge was significantly associated with contraceptive uses. Participants that had a high score on the SRH quiz (> 2) were more likely to report uses of contraception (OR = 1.35, 95%CI: 1.14–1.60). Male students with SRH knowledge score > 2 was more likely to use condom (OR = 1.90, 95%CI: 1.52–2.37).

### Reasons for contraceptive choice

The reasons of choosing different methods were presented in Table 5. Among participants choosing to use oral/injectable contraceptives, male students prioritized sexual partner’s request (49%), efficacy (47%), and practicality (36%), while female students focused more on the practicality (49%), the efficacy (44%) and having few side effects (36%). Participants choosing to use condoms and ECs, regardless of gender, focused more on the efficacy (65% vs. 64%; 58% vs. 56%), having few side effects (64% vs. 70%; 53% vs. 56%), and the practicality (58% vs. 59%; 51% vs. 69%).

**Table 5:** Reasons for choosing different contraceptive methods (%)

	Oral/injectable contraceptive^b^				Condom^b^				ECs^b^			
	male	female	χ2	Pr^a^	male	female	χ2	Pr^a^	male	female	χ2	Pr^a^
	*n* = 101	*n* = 55			*n* = 1122	*n* = 956			*n* = 141	*n* = 70		
Sexual partner’s request	49	33	3.62	0.06	26	15	38.59	< 0.01	26	24	0.07	0.79
Accessibility	22	24	0.07	0.79	23	19	5.01	0.03	23	16	0.95	0.33
Affordability	21	20	0.01	0.91	12	8	9.43	< 0.01	15	7	2.02	0.16
Practicality	36	49	2.67	0.10	58	59	0.09	0.76	51	69	3.94	0.05
Efficacy	47	44	0.12	0.73	65	64	0.39	0.53	58	54	0.22	0.64
Few side effects	27	36	1.57	0.21	64	70	8.33	< 0.01	53	56	0.10	0.75
Little influence on sexual sensation during intercourse	15	20	0.68	0.41	13	10	3.48	0.06	17	26	1.34	0.25
Others	1	4	1.32	0.25	1	1	0.65	0.42	2	4	0.55	0.46

^a^Pr for chi-square test

^b^Students who reported specific contraceptive use during last sexual intercourse were asked to provide reasons for contraception

We also investigated the reasons for not using contraception among participants who chose ‘no methods’ in the survey, and the results were shown in Additional file 2. Male students reported reasons like ‘on sexual partners’ requests’ (35%), ‘no need’ (27%), and ‘poor sexual experience’ (23%). Most female students reported reasons like: ‘no need’ (54%), ‘on sexual partners’ requests’ (21%), or ‘inaccessibility’ (15%).

## Discussion

The present study illustrated several important findings. First, condoms, oral contraceptives and ECs were the most known methods among Chinese college students. Most students reported using condom or withdrawal during the last sexual intercourse. Second, tobacco use and obtaining low SRH score in the SRH quiz were negatively associated with participants’ uses of contraceptives, while suburban residency, high monthly expenditure, high parental level of education, light alcohol consumption and high SRH score were positively associated with male and female participants’ uses of contraceptives. Third, Chinese college students generally prioritized the practicality, the efficacy, and having minimal side effects when choosing among different contraceptive methods.

This study showed that condoms, oral contraceptives and ECs were the most known methods in both male and female students. This result differs from previous studies conducted in Beijing, China [5, 12]. A study in 2000 showed that the most known methods were IUD, condoms, and oral contraceptives (61, 59, and 57%) among college students in Beijing [12]. Another study in 2012 revealed that condoms, oral contraceptives, and IUDs became the most commonly known contraceptive method (94, 90, and 62%) [5]. One reason for such differences over the years may be the diversification in sources of SRH knowledge. In 2002, the main sources of sexual knowledge were friends (36%) and books (20%). Other sources such as school-based sexual education, parents, and the Internet only accounted for a small part [13]. In recent years, Chinese college students reported that they attained sexual knowledge more often from the Internet (73%), health-related publications (60%), school-based education (28%), and birth control counselling (10%) [9]. Moreover, it is noticeable that the awareness of IUDs decreased a lot over the last decade. The decrease may be ascribed to China’s one-child policy. Chinese one-child policy started from 1980 and ended in 2015. During this period of time, a couple was only allowed to give birth to one child. In order to achieve the desired family planning goals, the central government encouraged the use of IUDs as a substitute to female sterilization in some areas [10]. Therefore, the prevalence of IUDs kept increasing and reached about 48% among women aged 20–49 in 2010. In some provinces such as Liaoning and Chongqing, the prevalence of IUDs was over 75% [10]. Recently, with the release of two-child policy, IUDs were no longer promoted by the government and thus both the prevalence and awareness of IUDs decreased.

Additionally, the present study revealed differences in the awareness of contraceptive methods between male and female students. Females were more aware of oral contraceptives, ECs and IUDs, while males were more aware of withdrawal and rhythm method. A study in 2012 found that the awareness of different kinds of contraceptive methods among females was relatively low compared to that among males in China [5]. However, the present study might reveal an increasing trend of female students’ contraceptive knowledge in recent years. This finding also resonated with a Korean study, indicating that the contraceptive knowledge were different between the two sexes, as students were inclined to use contraceptive methods with which they themselves could implement [14].

The associations of socioeconomic and lifestyle factors with contraceptive uses were also observed in the present study. First, female participants from suburban areas were more likely to use contraceptive methods in general. However, this association becomes no longer significant when stratified by different contraceptive methods. Previous studies showed that undergraduates from rural areas had lower SRH knowledge scores and held more conservative attitudes toward sexual behaviours [15]. Further studies are needed to identify how suburban residency influence students’ contraceptive use.

Second, higher monthly expenditure was positively associated with contraceptive uses, which was consistent with a Nigerian study, since students with a higher allowance were more likely to afford contraceptives [16].

Third, lifestyle factors, such as tobacco use and alcohol consumption, showed significant associations with condom uses. Smoking was negatively associated with condom use, while alcohol consumption presented a positive association. Previous studies have illustrated that among undergraduates, tobacco use was closely related to unhealthy lifestyles, such as poor personal hygiene, drug abuse, violence, and risky sexual behaviours [17, 18]. As young adults made the transition from high schools to universities, they might gain more independence and autonomy. However, many young adults still lacked self-discipline and self-protection, which might lead to risky behaviours [19, 20]. Therefore, tobacco use possibly revealed the insufficient behavioural guidance from schools. Insufficient behavioural guidance from schools in turn enhanced the possibility of developing risky sexual behaviour.

In terms of alcohol consumption, previous studies showed that binge drinking was negatively associated with condom use [20–22]. However, this study found that alcohol consumption was positively associated with condom use, especially among male students with light alcohol consumption. One possibility is that in this study, it was drinking habits that we inquired about, not the degree of soberness during sexual experience. A study in 2006 pointed out that the effect of drinking on behaviours was uncertain as it depended on various factors governing individual’s behaviours and beliefs [23]. Therefore, it was possible that regular and slight alcohol intake did not necessarily discourage condom use. Additionally, it should be noticed that oral/injectable contraceptives were long-term methods which required regular use. Thus, these two methods were not likely to be influenced by drinking habit. Meanwhile, the results showed that drinking was positively associated with the use of ECs among females. But the association was negative among males. A possible explanation is that ECs were remedial measures after intercourse. As EC implementers, females were more likely to remember using ECs, while males might be unaware of partners’ use of ECs.

Fourth, sexual knowledge was a crucial determinant of contraceptive use. Previous studies also indicated that higher SRH scores were significantly related to contraceptive uses [24]. Students with higher SRH scores were more likely to practice contraception than those with lower scores. The results resonated with other studies, showing that inadequate sexual knowledge was likely to deliver false information and discourage students’ contraceptive behaviours [25].

In the present study, students preferred contraceptive methods that were of high practicality, high effectiveness, and little side effects. In comparison, a 2015 study covering 49 Chinese universities found that the safety, the effectiveness, and the accessibility were the primary considerations for choosing contraceptive methods among Chinese students [8].

This study covered a relatively large population from eastern, central and western China, with a response rate of 89%, hence the results were likely to be representative of most Chinese college students. In the analysis, we investigated the awareness and the preferences for different contraceptive methods, and the reasons for using a specific type. The study has several limitations. First, the cross-sectional nature of the data reveals the possible associations between various contraceptive uses and the socioeconomic and lifestyle factors rather than causations. Prospective studies are needed to confirm possible causality. Second, information bias might exist, as some questions in the survey were personal and sensitive, but the anonymity of the survey could ameliorate such effect to some extent. Third, this study did not include marital status into the model because only 111 people (3.2%) reported having been engaged or married in the sample population. Since marital status is an important determinant in reproductive health, further investigation is needed. Fourth, selection bias may exist during the data collection when school-based contact persons used convenience samples. This might undermine the representativeness of the sample.

## Conclusions

The present study investigated the use and the awareness of contraceptive methods among Chinese college students. Chinese college students were mostly aware of condoms, oral contraceptives, and ECs. Meanwhile, Chinese college students most frequently used condoms and withdrawal during their last sexual intercourse among all contraceptive methods. Tobacco use, and low SRH score were negatively associated with uses of contraceptives, while high monthly expenditure, high parental level of education, light alcohol consumption, and high SRH score were positively associated with uses of contraceptives. Also, Chinese college students generally prioritized the practicality, the effectiveness and having few side effects when choosing which contraceptive methods to use. The present study reiterated the importance of contraceptive knowledge and uses among undergraduate students in China. Educators and organizations should design sexual education curriculum that is tailored to the need of college students of various demographic, socioeconomic and lifestyle backgrounds. Counselling office are needed to provide readily available information and any necessary assistance on issues related to sexual and reproductive health.

## Supplementary information

**Additional file 1:.** Appendix 1 Classification of alcohol consumption.

**Additional file 2:.** Appendix 2 Reasons for not using contraceptive methods (%).

**Additional file 3:.** Appendix 3 Logistic regression models examining the association of withdrawal methods and lifestyle factors.

## Data Availability

The data are available from the corresponding author on reasonable request.

## Abbreviations

SRH: Sexual and Reproductive Health
STIs: Sexually Transmitted Infections
GDP: Gross Domestic Product
HIV: Human Immunodeficiency Virus
AIDS: Acquired Immune Deficiency Syndrome
SD: Standard Deviation
ECs: Emergency Contraceptives
IUD: Intrauterine Device

## Footnotes

1 Oral emergency contraceptives refer to levonorgestrel tablets or similar medicine.
