# Supplementary material for: The socioeconomic and lifestyle determinants of contraceptive use among Chinese college students: a cross-sectional study
Source: Reprod Health. 2020 Aug 17;17:125. doi: 10.1186/s12978-020-00978-9 (PMC7433035; doi:10.1186/s12978-020-00978-9)
Supplement: Supplementary file 1 — Additional file 1:. Appendix 1 Classification of alcohol consumption. [file 12978_2020_978_MOESM1_ESM.docx]

Appendix 1 Classification of alcohol consumption

|  |  | Volume* | | |
| --- | --- | --- | --- | --- |
|  |  | 1 | 2 | 3 |
|  | several times a year | Light | Light | Moderate |
| Frequency | 1~3 times per month | Light | Moderate | Heavy |
|  | once a week | Moderate | Heavy | Heavy |

*1 referred to 0~50ml of Chinese white liquor, or 0~250ml of red wine, or 0~600ml of beer

2 referred to 50~100ml of Chinese white liquor, or 250~500ml of red wine, or 600~1200ml of beer

3 referred to more than 100ml of Chinese white liquor, or more than 500ml of red wine, or more than 1200ml of beer
