# Supplementary material for: The socioeconomic and lifestyle determinants of contraceptive use among Chinese college students: a cross-sectional study
Source: Reprod Health. 2020 Aug 17;17:125. doi: 10.1186/s12978-020-00978-9 (PMC7433035; doi:10.1186/s12978-020-00978-9)
Supplement: Supplementary file 2 — Additional file 2:. Appendix 2 Reasons for not using contraceptive methods (%). [file 12978_2020_978_MOESM2_ESM.docx]

Appendix 2 Reasons for not using contraceptive methods (%)

|  | Males(%) | Females(%) | Pr* |
| --- | --- | --- | --- |
|  | n=104 | n=52 |  |
| Sexual partner's request | 35 | 21 | 0.08 |
| No need | 27 | 54 | <0.05 |
| Poor sexual experience | 23 | 13 | 0.16 |
| Inaccessibility | 14 | 15 | 0.87 |
| Difficult to use | 8 | 4 | 0.36 |
| Others | 6 | 8 | 0.64 |
| Financial difficulty | 5 | 2 | 0.38 |
| Many side effects | 5 | 10 | 0.25 |
| Inefficacy | 0.00 | 4 | <0.05 |

*Pr for chi-square test
