# Supplementary material for: The socioeconomic and lifestyle determinants of contraceptive use among Chinese college students: a cross-sectional study
Source: Reprod Health. 2020 Aug 17;17:125. doi: 10.1186/s12978-020-00978-9 (PMC7433035; doi:10.1186/s12978-020-00978-9)
Supplement: Supplementary file 3 — Additional file 3:. Appendix 3 Logistic regression models examining the association of withdrawal methods and lifestyle factors. [file 12978_2020_978_MOESM3_ESM.docx]

|  | Withdrawal | | | |
| --- | --- | --- | --- | --- |
|  | Male (n^a^=293) | | Female(n^a^=294) | |
|  | OR | 95% CI | OR | 95% CI |
| Tobacco use |  |  |  |  |
| No smoking | 1.00 |  | 1.00 |  |
| 1-10 cigarettes/day | 0.90 | 0.65-1.25 | 1.73 | 0.94-3.18 |
| >10 cigarettes/day | 0.72 | 0.43-1.20 | 1.52 | 0.50-4.69 |
| *P for trend* | 0.77 | | 0.24 | |
| Alcohol consumption |  |  |  |  |
| Abstinence | 1.00 |  | 1.00 |  |
| Light | 1.14 | 0.67-1.95 | 0.77 | 0.56-1.06 |
| Moderate | 1.40 | 0.77-2.54 | 0.75 | 0.37-1.54 |
| Heavy | 1.17 | 0.61-2.24 | 0.54 | 0.16-1.77 |
| *P for trend* | 0.58 | | 0.65 | |

Appendix 3 Logistic regression models examining the association of withdrawal methods and lifestyle factors

a. The n refers to the number of participants who reported using withdrawal method during the last sexual intercourse.

b. Adjustments: type of school, school, geographical distribution, expenditure per month, hometown, parent education, tobacco use, alcohol consumption, sex education.
